# Lifestyles Under Lockdown: A Scoping Review of International Studies on Type 2 Diabetes Self-Management Behaviors During COVID-19

**DOI:** 10.3389/fcdhc.2022.830353

**Published:** 2022-03-15

**Authors:** Caroline Cummings, Kagnica Seng, Ryan Tweet, Julie Wagner

**Affiliations:** ^1^ Department of Psychological Sciences, Texas Tech University, Lubbock, TX, United States; ^2^ Department of Psychological Sciences, Central Connecticut State University, New Britain, CT, United States; ^3^ Division of Endocrinology, Diabetes and Clinical Nutrition, Oregon Health & Science University, School of Medicine, Portland, OR, United States; ^4^ Division of Behavioral Sciences and Community Health, University of Connecticut School of Dental Medicine and Department of Psychiatry, School of Medicine, Farmington, CT, United States

**Keywords:** type 2 diabetes, COVID-19, self-management, physical activity, diet, adherence

## Abstract

**Background:**

The effect of the COVID-19 pandemic on diabetes self-management behaviors is unclear.

**Objectives:**

This paper is a scoping review of studies examining health behaviors among people with type 2 diabetes during the COVID-19 pandemic.

**Eligibility Criteria:**

We searched articles available in English using the Search terms “COVID” and “diabetes”, and, separately, each of the following terms: “lifestyle”, “health behavior”, “self-care”, “self-management”, “adherence”, “compliance”, “eating”, “diet”, “physical activity”, “exercise”, “sleep”, “self-monitoring of blood glucose”, or “continuous glucose monitoring”.

**Sources of Evidence:**

We searched PubMed, PsychInfo, and Google Scholar databases from December 2019 through August 2021.

**Charting Methods:**

Data were extracted by 4 calibrated reviewers and study elements were charted.

**Results:**

The search identified 1,710 articles. After screening for relevance and eligibility, 24 articles were included in this review. Findings show the strongest evidence for reduced physical activity and stable glucose monitoring and substance use. There was equivocal evidence for deleterious changes in sleep, diet, and medication intake. With one minor exception, there was no evidence for favorable changes in health behaviors. Limitations of the literature include small samples, predominantly cross-sectional study designs, reliance on retrospective self-reports, sampling through social media, and few standardized measures.

**Conclusions:**

Early studies of health behaviors among people with type 2 diabetes during the COVID-19 pandemic suggest a need for novel interventions to support diabetes self-management, especially targeting physical activity. Future studies should go beyond documenting changes in health behaviors and examine predictors of change over time.

## Introduction

Over 460 million individuals, which equates to over six percent of the global population, have type 2 diabetes mellitus (T2DM) (1). Prevalence rates continue to rapidly incline across age groups and all regions of the world (1). The diabetes epidemic is alarming, as a diagnosis of T2DM is associated with a greater risk of mortality (2) and chronic (e.g., heart disease; renal disease) (3) and terminal (e.g., cancer) illnesses (4). To effectively manage T2DM, patients are prescribed a comprehensive self-management plan (5). Diabetes self-management plans typically encourage regular engagement in various health behaviors, including but not limited to self-monitoring blood glucose levels (SMBG), medication intake, conducting foot examinations, eating a balanced diet, and engaging in physical activity (5). However, due to various factors (e.g., financial constraints, difficulties with planning, low self-efficacy), patients with T2DM often struggle with regularly completing their diabetes self-management behaviors (6).

In response to increased stressors and social distancing requirements, the Coronavirus Disease of 2019 (COVID-19) pandemic has affected individuals’ engagement in daily health behaviors (7–9). It can be hypothesized that health behavior engagement in the T2DM community has been impacted as well. For example, in a pre-pandemic study, adults with T2DM reported financial strain as a reason for below-target medication intake (6). The pandemic has affected the financial security of many individuals globally, causing downstream effects on financial stress and psychopathology (10), such that it can be anticipated that patients with T2DM have encountered additional barriers that further reduced their medication intake in the context of the COVID-19 pandemic. Accordingly, researchers have examined changes in metabolic outcomes in patients with T2DM during the COVID-19 pandemic (11–15). Data are equivocal and suggest metabolic control has either decreased or remained stable in patients with T2DM during the pandemic (11–15). Yet, less attention has been paid to examining changes in the diabetes self-management behaviors that are important in contextualizing metabolic outcomes in this population. The pandemic is expected to persist (16) and pandemics will continue to present in the future (17). It is imperative to explore diabetes self-management behavior engagement during the COVID-19 pandemic to identify ways to support patients with T2DM, both during the pandemic and beyond.

The purpose of the current review was two-fold: 1) to characterize diabetes self-management behavior engagement in individuals with T2DM during the COVID-19 pandemic, and 2) where available, to characterize changes in diabetes self-management behavior engagement in individuals with T2DM from the pre-pandemic period to the pandemic period. Given the dearth of studies in the area, we conducted a scoping review. Scoping reviews are uniquely indicated to identify and map types of available evidence, to examine how research has been conducted, and to identify knowledge gaps. Based on the extant literature reviewed, we provide recommendations for future research and preliminary suggestions for practice with individuals with T2DM during and after the pandemic.

## Methods

### Search

We followed methodological guidance (18) for Preferred Reporting Items for Systematic reviews and Meta-Analyses extension for Scoping Reviews (PRISMA-ScR) (19). We systematically searched PubMed, PsychInfo, and Google Scholar databases from December 2019 through August 2021. We restricted the search to articles available in English and used the Search terms “COVID” and “diabetes”, and, in separate searches, each of the following terms: “lifestyle”, “health behavior”, “self-care”, “self-management”, “adherence”, “compliance”, “eating”, “diet”, “physical activity”, “exercise”, “sleep”, “self-monitoring of blood glucose”, “SMBG”, “continuous glucose monitoring”, or “CGM”.

### Charting

One author (JW) drafted a data extraction chart based on PRISMA scoping guidelines which was beta tested by all four authors and modified to include directional arrows for results. Data were charted for author, publication year, country and time period of data collection, sample size and characteristics, sampling method, health behavior(s) studied, specific measures, results, and unique or ancillary findings.

### Reviewer Calibration

One author (KS) conducted the electronic database searches and screened all titles and abstracts to remove irrelevant papers. Two screeners (KS and JW) reviewed remaining abstracts to remove ineligible papers. All four authors (CC, RT, KS, JW) read the remaining full articles, and further removed ineligible papers. To address consistency across the reviewers and to decrease bias, five articles were reviewed independently by all four reviewers and extracted data were compared. Differences in responses were noted and discussed until consensus was reached. An additional five articles were reviewed independently by two reviewers (CC and RT) using the same process until the first author (CC) judged that data extraction was sufficiently consistent. The first author reviewed the final tables for accuracy and consistency.

### Data Synthesis

We followed guidelines for conducting (18) and reporting (19) a scoping review. A scoping review is a particular type of review that is indicated for topics in which the body of literature is nascent. A scoping review should be differentiated from, for example, a systematic review or a meta-analysis, which are indicated when a body of literature is well developed and are designed to produce statements to guide clinical decision making (20). Scoping reviews do not report indicators of assessments of bias. Also, scoping reviews are, by definition, not designed to produce clear practice guidelines, but instead to update readers regarding needed future research. To describe the role of COVID-19 on health behaviors, attempts were made to characterize results in terms of increase, decrease, or no change in the health behavior compared to pre-COVID-19 levels.

## Results

**Figure 1:**
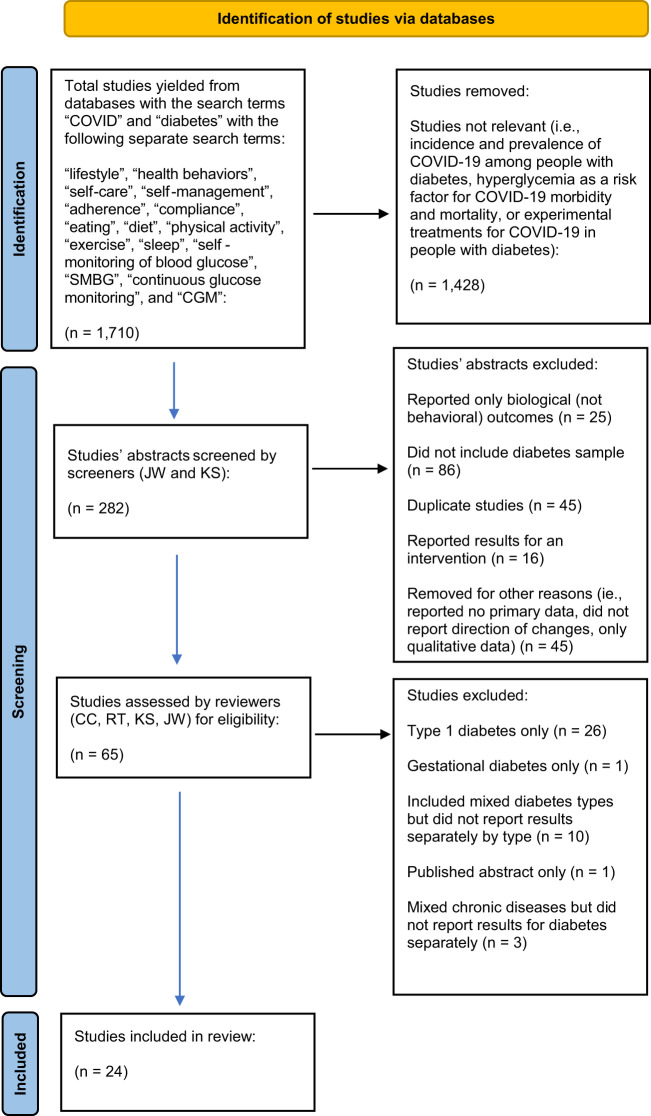
PubMed, PsychInfo, and Google Scholar databases from December 2019 through August 2021.Page MJ, McKenzie JE, Bossuyt PM, Boutron I, Hoffmann TC, Mulrow CD, et al. The PRISMA 2020 statement: an updated guideline for reporting systematic reviews. BMJ 2021;372:n71. doi: 10.1136/bmj.n71.

See **Figure 1** for PRISMA-ScR flow diagram. Search results identified 1,710 articles. Of them, 1,428 articles were removed because they were not relevant. Many of the articles that were removed at this stage reported, for example, incidence and prevalence of COVID-19 among people with diabetes, hyperglycemia as a risk factor for COVID-19 morbidity and mortality, or experimental treatments for COVID-19 in people with diabetes.

The remaining 282 abstracts were reviewed and of them, n=25 were removed because they reported only biological (not behavioral) outcomes (to be included, articles were not required to report health behaviors as the primary outcome but were required to report health behaviors). Additional articles were removed because they were case studies or did not include a diabetes sample (n=86), reported results of an intervention (n=16), were reviews that did not report any primary data (n=42), included qualitative data (n=1), or reported that there had been changes in health behaviors but did not report the direction of the changes (n=2). Of the remaining articles, an additional n=45 were removed because they were duplicates across searches. The remaining full articles were read and an additional n=41 articles were removed because they reported data only for type 1 diabetes mellitus (n=26) or gestational diabetes (n=1), or they included mixed diabetes types but did not report results separately by type of diabetes (n=10). One article was a published abstract only and three reported mixed chronic diseases and did not report results for diabetes separately.

**Figure 2:**
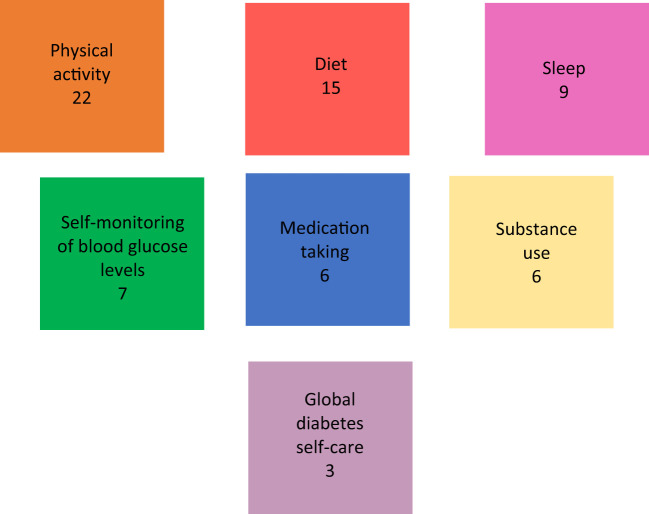
Map of health behaviors measured by number of studies.

Articles were divided into two categories based on study design. One category of studies asked participants about their current level of health behaviors; those articles only reported frequencies or means of health behaviors during COVID-19 (n=5). The other category attempted to characterize changes in self-management from pre-COVID-19 levels to COVID-19 levels (n=19). See **Figure 2** for a map of health behaviors measured by number of studies.

In the first category, articles reported data from Brazil, China, Ethiopia, and Turkey. Sample sizes ranged from n=101 in Turkey (21) to n=1,253 in China (22). All participants were recruited from clinics. Shimels et al. (23) studied people with diabetes and comorbid hypertension. For the articles that reported a time period of data collection, dates ranged from March 2020 to August 2020. See **Table 1**. Note that where articles reported only frequencies—even if there was considerable variability—for parsimony we present only the modal response.

**Table 1:** Frequency of health behaviors among people with type 2 diabetes during COVID only.

	Author, year +	Sample size, age range, diabetes type, and place	Health behaviors studied	Specific measures++ and mode of data collection (internet, phone call, mail-in survey)	Sampling (community; clinic; registry; social media)	Data collection cross- sectional or longitudinal	Month and year of data collection	Main findings	Notes
1	(24) Alessi et al., 2020	N=120; Adults; mixed (T2DM n=68 type 2); Brazil	Diet; sleep	26-item Eating Attitudes Test (EAT; 25) and 10-item Mini Sleep Questionnaire (26); phone survey	Outpatient endocrine clinic of a public hospital	Cross-sectional	8-day period; dates not reported	Modal responses: 74% screened positive for an eating disorder; 78% screened positive for sleep disorder	Diabetes distress and screens for other psychiatric disorders also reported
2	(27) Kaplan Serin & Bulbuloglu, 2021	N=103; Adults; T2DM; Turkey	Diet; PA	Diabetes Self-Management Questionnaire (DSMQ; 28); in person survey	University internal medicine clinic	Cross-sectional	Unknown	Modal responses: Irregular PA; high dietary compliance	Working more days outside the home predicted higher self-care. Fear of COVID and death reported.
3	(21) Onmez et al., 2020	N=101; Adults; T2DM; Turkey	SMBG; PA; diet	Clinical forms and Short Form 36-item survey (29, 30); in person survey	University diabetology clinic	Cross-sectional	July-August 2020	Modal response: SMBG rarely occurred; PA rarely or never occurred; diet rarely occurred.	Comparisons of A1c, glucose, and BMI from pre-lockdown to lockdown reported.
4	(23) Shimels et al., 2021	N=409; Adults; T2DM and comorbid hypertension; Ethiopia	Medication adherence	Morisky Medication Adherence Scale (MMAS; 31), in-person survey	7 public health facilities in the capital city	Cross- sectional	August 2020	Modal response: low adherence to anti-diabetes and anti-hypertension medications	Higher income, comorbidities and substance use predicted non-adherence. Data provided about sleep and access to medications
5	(22) Tao et al., 2020	N=1253; Adults; mixed (T2DM n=1159); China	Diet; PA; medication adherence; SMBG; foot self-examination	Health behavior questionnaire; phone survey	Patients with past-year hospital discharge	Cross-sectional	March-April 2020	Modal response: SMBG >2x/week, no foot self-examination, no smoking; high medication adherence, regular meal intake, and moderate PA.	T1DM data reported separately; factors linked to glycemic outcomes included

Three studies assessed physical activity. Onmez et al. (21) found that the modal response was that physical activity rarely or never occurred. Kaplan Serin and Bulbuloglu (27) found that physical activity was irregular. Tao et al. (22) found moderate levels of physical activity.

Four studies assessed diet. Onmez et al. (21) found that the modal response was that following dietary recommendations rarely occurred. Alessi et al. (24) found that 74% screened positive for an eating disorder. However, Kaplan Serin and Bulguloglu (27) found high dietary compliance and Tao et al. (22) found regular meal intake.

One study (24) assessed sleep and found that 78% screened positive for a sleep disorder.

Two studies assessed medication intake. Shimels et al. (23) found low levels of medication intake and Tao et al. (22) found high levels of medication intake.

Two studies assessed SMBG. Onmez et al. (21) found that the modal response was that SMBG rarely occurred. Tao et al. (22) found that the modal response was ≥ 2 times per week for SMBG.

No studies in this category assessed substance use.

In the second category (i.e., papers that described change in health behaviors from pre-COVID-19 to COVID-19), articles reported data from Australia, Brazil, France, India, Japan, Malaysia, Mexico, the Netherlands, Poland, Saudi Arabia, Spain, Turkey, United Kingdom, and USA. Notably, one study examined bariatric surgery patients (32) and another studied children and adolescents (33). Only n=3 implemented longitudinal methodology, i.e., followed the same cohort over time; they were based in India (34), the United Kingdom (35), and Australia (36). For the articles that reported a time period of data collection, dates ranged from March 2020 to April 2021, though Rowlands et al. (35) had baseline data dating back to 2017. One study in Brazil compared data from a sample of people with diabetes during COVID-19 to data from an age-matched sample of people with diabetes in 2016 (37). The remainder (n=15) of studies asked participants to report their current health behaviors and also to recall their pre-COVID-19 health behaviors. Sample sizes ranged from n=56 in Japan (38) to n=1,633 in Brazil (39). N=4 were recruited using social media, n=3 reported data from people with diabetes in registries or who were already enrolled in research studies, and the remainder (n=12) recruited clinic samples. See **Table 2**.

**Table 2:** Change in health behaviors from pre-COVID period to COVID among people with type 2 diabetes.

	Author, year +	Sample size, age range, diabetes type, and place	Health behaviors studied	Specific measures++ and mode of data collection (internet, phone call, mail-in survey)	Sampling (community; clinic; registry; social media)	Data collection cros-sectional or longitudinal	Month and year of data collection	Main findings	Notes
1.	(40) Alshareef et al., 2020	N=394; Adults; T2DM; Saudi Arabia	Medication taking; SMBG; PA; diet	8-item health behavior questionnaire; phone survey	National guard primary care clinics	Cross-sectional	Not reported	Modal responses: *↓ medication taking compliance; ↓ PA and diet ; → SMBG	Psychological distress also reported
2.	(39) Binhardi et al., 2021	N=1633; Adults; mixed (T2DM n=628); Brazil	Alcohol consumption	Alcohol consumption questionnaire; internet survey	Social media; traditional media (e.g., radio, television)	Cross-sectional	September-October 2020	Modal responses: No alcohol consumption or → alcohol consumption; high consumption of fatty food and sweets, and low PA all >40%.	Participants also reported changes in glucose control and COVID status; data from T1DM patients also reported
3.	(33) Cheng et al., 2021	N=123; Children and adolescents; mixed (T2DM n=30); Malaysia	PA; diet; sleep; screen time	Physical Activity Questionnaire (PAQ; 41, 42); health behavior questionnaire; in-person paper-and-pencil survey	Hospital pediatric diabetes clinic	Cross-sectional	June-December 2020	*↑ sleep and screen time; ↓ PA and meal frequency	Results for boys and girls reported separately; body mass index and glycemia also reported
4.	(43) Fisher et al., 2020	N=1382; Adults; mixed (T2DM n=619); USA	Diet; PA; medication taking; SMBG; reviewing BG trends	7-item health behavior questionnaire; internet survey	Diabetes research registry	Cross-sectional	April 2020	Modal responses: ↑food intake; ↓ PA; → medication taking, SMBG, and reviewing BG trends	Data about stress, isolation, and impact on access to care and supplies reported
5.	(37) Franco et al., 2021	N=260; Adults; mixed (T2DM n=150); Brazil	Overall diabetes self-care	Portuguese version of Self-Care Inventory Revised (SCI-R; 44); phone survey	Endocrinology outpatient clinic	Cross-sectional with comparison group from 2016	1 month after start of social distancing guidelines; dates not reported	→ SCI score	Data from T1DM patients also reported
6.	(45) Ghosh et al., 2020	N=150; Adults; T2DM; India	Diet; PA; SMBG; sleep; substance use	40-item health behavior questionnaire; phone survey	Clinic	Cross sectional	May 2020	Modal responses: → SMBG, sleep, appetite, quantity or quality of diet; ↓ PA; no alcohol or tobacco	87% reported “mental stress”
7.	(46) Grabia, et al., 2020	N=124; adults and children; mixed (T2DM n=34); Poland	PA; diet; sleep; screen time	Health behavior questionnaire; internet survey	Social media diabetes groups	Cross-sectional	July 2020	Various favorable and unfavorable changes in diet and PA	Most health behavior changes not reported by diabetes type. Detailed food and exercise items.
8.	(47) Hansel et al., 2021	N=5280, Adults, mixed (T2DM and BMI ≥ 25 n=2632); France	Diet; snacking; PA; substance use	Health behavior questionnaire; web application survey	Social media/web application	Cross-sectional	March-April 2020	Modal responses: → overall dietary intake, snacking, smoking, vegetable, fruit consumption, and alcohol consumption; ↓ PA	Data about diabetes located in supplemental data files. Higher BMI predicted healthier behavior changes
9.	(38)Hasegawa et al., 2021	N=56; Adults; T2DM; Japan	Sleep; exercise	Health behavior questionnaire; mode of data collection not reported	Clinic	Retrospective cohort study	April-May 2020	Modal responses: ↓ PA; either → or ↑sleep duration	n=22 reported health behaviors; data extracted from larger study of skeletal muscle mass; reduced sleep and exercise predicted loss of muscle mass
10.	(48) Munekawa et al., 2021	N=183; Adults; T2DM; Japan	Sleep; PA; diet	6-item health behavior questionnaire; in-person survey	University endocrinology clinic	Cross-sectional	April-May 2020	Modal responses: ↓ PA; → sleep, total diet intake, snack intake	Higher stress was associated with higher consumption of prepared food and lower exercise
11.	(34) Rastogi et al., 2020	N=422; Adults; T2DM; India	PA	Global Physical Activity Questionnaire (49, 50); pre-lockdown: in-person survey, during lockdown: phone survey	Diabetes clinic	Longitudinal	Not reported	*↑ PA	Increased self-reported PA was not associated with changes in weight or glycemia
12.	(51) Regeer et al., 2021	N=536; Adults; T2DM; Netherlands	PA	Physical activity questionnaire; Short Questionnaire to Assess Health Enhancing PA (SQUASH; 52); internet survey	Registry of participants of previous PA research in past 5 years	Cross-sectional	May 2020	Modal response: → PA	Higher emotional wellbeing was associated with decreased likelihood of becoming less active
13.	(35) Rowlands et al., 2021	N=165; Adults; T2DM; United Kingdom	PA; sleep	Wrist accelerometer	Registry of participants of previous chronotype and glycemia study	Longitudinal	Baseline: 2017-2020; COVID: May to June 2020	*↓ overall PA, days per week with 30 and 60 minutes of MVPA; *↑ inactive minutes; → sleep duration, MVPA minutes, continuous MVPA for 10 mins and 30 mins	Being a woman, older, or an ethnic minority was associated with lower PA
14.	(53) Ruiz-Roso et al., 2020	N=72; Adults; T2DM; Spain	Diet; PA; food craving	Food Frequency Questionnaire (54), International Physical Activity Questionnaire (55, Food Craving Questionnaire (56); phone survey	Clinic	Cross-sectional	April-May 2020	↑* dairy, vegetable, snack, and sugary food intake; ↓* PA	HbA1c noted
15.	(36) Sacre et al., 2021	N=489; Adults; T2DM; Australia	PA; sedentary time; alcohol intake; medical visits; SMBG; medication adherence	Confidence in Diabetes Self-Care (57) and other health behavior questionnaires; phone/internet survey	Clinic and registry	Longitudinal population-based cohort	April-May 2020	Trend for ↓ PA; → alcohol intake, SMBG, and medication adherence; ↑* medical visits	Planned walking replaced incidental walking. Symptoms of anxiety, depression, and diabetes distress also reported.
16.	(58) Sankar et al., 2020	N=110; Adults; T2DM; India	SMBG; PA; dietary adherence; food intake; and sleep	7-10min questionnaire; verbal, in-person survey	Hospital outpatient diabetes clinic	Cross-sectional	May-June 2020	Modal response: ↑ vegetable and fruit intake; ↓ snacks, fried, or processed foods intake; → PA, sleep, SMBG, and dietary adherence	Higher stress and anxiety related to worsened sleep and diet habits. Decreased access to medications; A1c noted.
17.	(59) Silva Tinoco, et al., 2021	N=212; Adults; T2DM; Mexico	Global self-care	Summary of Diabetes Self-Care Activities; unknown mode of data collection	Patients of a quality improvement program at a diabetes pharmacy	Cross-sectional	April-May, 2020 with comparison group from February-March 2020	*↓global self-care behavior (diet, PA, SMBG)	Higher pre-pandemic self-care scores predicted fewer self-care problems during pandemic
18.	(60) Utli et al., 2021	N=378; Adults; T2DM; Turkey	PA; substance use; sleep	Diabetes Self-Management Questionnaire (DMSQ; 28); paper-and-pencil, in-person survey	Outpatient endocrine clinic in a government hospital	Cross sectional	December 2020 to April 2021	Modal responses: COVID negatively impacted diabetes self-management; ↓ sleep quality and PA; no smoking	Data on demographic and psychosocial factors impacting diabetes self-management reported
19.	(32) Wysocki et al., 2020	N=885; Adults; Pre- and post-operative bariatric surgery patients with and without T2DM (T2DM=356); Poland	Diet; PA	Health behavior questionnaire; internet survey	Social media	Cross-sectional	Not reported	Modal responses: ↓ PA in both pre-operative and post-operative patients with T2DM	Bariatric care under COVID reported

For studies in which frequencies are reported, for parsimony, we report only the modal response though there may have been considerable variability in responses.

T1DM, type 1 diabetes mellitus; T2DM; type 2 diabetes mellitus; PA; Physical activity; SMBG, self-monitoring of blood glucose; CGM, continuous glucose monitor.

→ = no change.

↓ = decrease.

↑ = increase.

* = change reported as significant.

Social media includes, for example, Facebook, Instagram, Twitter.

Conversation Apps includes, for example, What’sApp, Telegram.

Traditional media are, for example, radio, television, newspaper.

Sixteen of the 19 articles assessed physical activity. The majority (n=15) of studies reported decreased physical activity (32, 33, 35, 38, 40, 43, 45–48, 53, 60) or low physical activity (39). Three studies reported no change (36, 51, 58) and one reported increased physical activity (34). Regeer et al. (51) found that changes in physical activity were related to stress and emotional wellbeing; participants with higher emotional wellbeing were more likely to report no change than to report decreased physical activity. Rowlands et al. (35) found that being a woman or having a higher body mass index predicted lower physical activity and higher inactive time. Being older or an ethnic minority predicted higher inactive time.

Eleven studies assessed diet. Several studies reported an increase in unfavorable dietary habits, including decreased dietary adherence (40), high consumption of fatty foods and sweets (39), and increased food intake (43, 53). One study reported favorable changes in diet, i.e., increased fruit and vegetables and decreased snacks, fried, or processed foods (58). Four studies reported no change in dietary habits, including no change in appetite, quantity or quality of diet, or consumption of fruits (45), no change in overall dietary intake, snacking, fruit and vegetable consumption (47), and no change in total dietary intake or snack intake (48). One study reported various detailed favorable and unfavorable changes in diet (46).

Eight studies reported on sleep. Cheng et al. (33) reported increased sleep and Hasegawa et al. (38) reported increased sleep duration. One study reported decreased sleep quality (60). Four studies reported that the modal response was no changes in sleep (35, 45, 48, 58).

Four studies reported medication intake. Two studies reported decreased medication intake (40), and two studies reported no change (36, 43).

All five studies that assessed SMBG reported no change (36, 40, 43, 45, 58).

Six studies reported substance use. For alcohol, Binhardi et al. (39) and Ghosh et al. (45) reported that modal responses were no alcohol consumption or no change in alcohol consumption, respectively. Hansel et al. (47) and Sacre et al. (36) reported no change in alcohol consumption. For smoking, Utli et al. (60) and Ghosh et al. (45) reported no smoking as the modal response.

Three studies examined global diabetes self-care scores. One study reported no change (37) and two studies (59, 60) reported decreased total diabetes self-care scores. Silva-Tinoco et al. (59) found that higher pre-pandemic self-care was related to fewer problems with self-care during the pandemic. Utli et al. (60) found that being a man, smoker, and older in age, and having more anxiety and stress and decreased support from healthcare providers, predicted lower global self-care.

## Discussion

This scoping review examined the extant literature regarding health behavior engagement among individuals with T2DM during the COVID-19 pandemic. Across studies, the strongest evidence was for a reduction in physical activity and there was small but consistent evidence for no change in SMBG and substance use. Global diabetes self-care decreased in two of three studies. Overall, dietary habits appear to have remained stable or worsened and in one study a high percentage of participants screened positive for a possible eating disorder. There were equivocal findings regarding medication intake and sleep. Older age, lower pre-pandemic self-care, and decreased emotional wellbeing and support from healthcare providers predicted greater deterioration in diabetes self-care behaviors. Unfortunately, many studies included the administration of unstandardized measures and descriptive statistics as an analytic plan; thus, a comparison of findings across studies and evaluation of effect sizes could not be conducted. This review points to multiple recommendations for future research and clinical practice with the T2DM population.

Most studies demonstrated a reduction in physical activity during the pandemic, highlighting the need for novel interventions to support individuals with T2DM in engaging in this essential diabetes management behavior. A few studies (35) pointed out that when activity occurred, planned fitness activities (e.g., a workout session) may have replaced incidental physical activity (e.g., walking to the store). It has been hypothesized that reductions in physical activity by the general public in the context of the pandemic might be a function of stress and fear associated with potential contraction of COVID-19 (7), which may in turn motivate individuals to reduce or avoid physical activity in groups and in public areas. This may be especially true for the T2DM population, who have received public health messaging that they are at a higher risk of contracting COVID-19 and experiencing severe symptoms due to their altered immune and metabolic system functioning (61). Given the key role of physical activity in supporting optimal glycemic control in this population (62), it is imperative to further investigate patterns of physical activity during the pandemic and as societies shift towards “normalcy” and a post-pandemic period.

A lack of change in SMBG levels was observed in the studies that reviewed glucose monitoring, which suggests that, despite an increase in stress and disruption in individuals’ daily routine (63), SMBG remained relatively stable in this population. Given that SMBG is strongly correlated with glycemic control (64), and some studies show an increase in blood glucose levels and glycated hemoglobin during the pandemic (11), this finding is surprising and suggests that other key diabetes management behaviors might be impacted during the pandemic that are causing downstream unfavorable effects on individuals’ glycemic control. Moreover, the lack of change in SMBG should be of concern when considering that those individuals who demonstrated low SMBG pre-pandemic were also likely to demonstrate continued difficulties with SMBG (65); thus, there remains a subpopulation of individuals with T2DM (i.e., those who demonstrate low SMBG and, in turn, above-target glycemic control) who are highly susceptible to experiencing adverse reactions when acquiring COVID-19. Continued study of SMBG and its relation to glycemic control, and possibly COVID-19 infection severity, across the pandemic is warranted.

Most individuals with T2DM reported little to no substance use during the pandemic and no change from the pre-pandemic period. This is inconsistent with data highlighting the widespread increase in substance use by the general population during the pandemic (66). It is possible that participants may have underreported substance use due to demand characteristics and social desirability, especially when questionnaires were administered by healthcare providers (e.g., studies with samples of patients that were being seen in clinic at the time of questionnaire completion). Nonetheless, findings suggest that substance use may be a relatively lower priority for researchers and clinicians to address in their work with people with diabetes, compared to other health behaviors that were more clearly impacted by the pandemic, such as physical activity.

Studies examining changes in and frequency of engagement in the remaining health behaviors yielded equivocal results. Specifically, there was a near-even split of studies that reported a lack of change or decrease in medication intake, sleep, and diet. Only two studies suggested a favorable increase in any of these health behaviors, finding an increase in participants’ consumption of dairy, vegetables, and/or fruits (53, 58), but this was an exception. For the T2DM population as a whole, the reviewed studies suggest that there are no consistent or compelling data to suggest that diabetes self-management behaviors improved during the pandemic.

There are several possible explanations for differing results across studies, including the time frame of data collection, region in which the study was conducted, measure/questionnaire employed, and sampling strategy. First, it is possible that contradictory findings may be a result of differences in COVID-19 guidelines and lockdown regulations across time and regions. For example, medication intake and SMBG may have been influenced by an individual’s inability to pick up prescriptions in regions that had more restrictive lockdowns, or when medical supply chains were disrupted. Second, inconsistent findings may have emerged due to use of varying measures. Most studies reviewed designed their own questionnaires which varied by content, item stem, and response options. Most were retrospective self-reports that required participants to recall temporally distal health behaviors from 6-months to 1-year ago or more. Self-report measures are subject to numerous sources of bias and retrospective measures are subject to poor recall. Depending on the research question at hand, objective measures may be preferable when feasible. Objective sleep and physical activity characteristics may be best captured by actigraphy data, which was only used in one study that indicated decreased overall physical activity and stable sleep duration from the pre-pandemic period to the pandemic period (35). Diet is extremely challenging to measure without intensive methods, such as photographic food records (67), which were not used in any studies. Measuring the frequency of glucose monitoring is made easier with uploadable meters and continuous glucose monitors, but these data were not reported in most studies. Also, substance use is best measured *via* daily reports (68) or, in some cases, biospecimen testing. Finally, sampling strategies may have yielded differing results. Several studies recruited participants through social media. Data from those studies may have limited generalizability, as reviews have shown that participants recruited through social media differ from those recruited through traditional methods (69).

Perhaps the greatest missed opportunity in the literature to date is that, with a few exceptions (35, 51, 59, 60), the majority of studies simply reported change in behavior as frequencies of ‘increase’, ‘decrease’, or ‘no change,’ but did not examine predictors of direction or degree of change. In order to efficiently direct resources to the patients most in need of self-management support, it is helpful to know the individual characteristics that predict diabetes self-management during the pandemic. For example, perhaps individuals who experienced financial downturn, who live alone, or whose pre-pandemic glycemic control was suboptimal are in the greatest need of self-management support during the pandemic. On the other hand, perhaps those who were allowed to work from home, with greater access to outdoor space for physical activity, or with home prescription delivery were able to maintain or even improve their diabetes self-management. Elucidating the factors related to behavioral vulnerability and resilience can help tailor intervention development and delivery.

It should be acknowledged that researchers had to move quickly to document changes in diabetes self-management during the COVID-19 pandemic. Planning the study, procuring funding, obtaining ethics approval, and collecting data are all time intensive. Moreover, this research was being conducted in the midst of social distancing measures that made best-practices for behavioral diabetes research difficult or impossible. Efforts to conduct research under these conditions are to be lauded. Notwithstanding these commendations, as researchers continue to collect data on pandemic-specific health behavior engagement in this population, it will be important going forward to use standardized, objective measures where possible, as well as recruit participants who are well characterized, follow them over time across different phases of the pandemic, and examine predictors of behavior change.

### Limitations

Our conclusions are qualified by several limitations of this review. First, database review concluded in August of 2021, and it is likely that additional articles have been published since then but were not included in the current review. Second, studies reviewed were limited to data from individuals with T2DM. Findings may not be representative of changes in health behavior in individuals with type 1 diabetes, gestational diabetes, or pre-diabetes. This is an area for future research. Third, most studies were conducted with adult samples, thus findings may not generalize to health behavior of children and adolescents with diabetes. Fourth, due to the extreme heterogeneity in samples, sampling, and measures across studies, a meta-analysis could not be conducted, thus the interpretability of findings is limited. Fifth, in those studies that reported frequencies only, we reported modal responses. Across every study, there were subsamples of participants who reported favorable changes, unfavorable changes, and stable health behaviors. Therefore, it should not be concluded that all individuals with T2DM demonstrated self-management decrements in response to the pandemic. Finally, scoping reviews do not report indicators of assessment of bias and do not produce clear practice guidelines.

### Conclusions and Future Directions

Findings from this brief review indicate that individuals with T2DM demonstrated reduced engagement in physical activity during the pandemic and no change in SMBG or substance use. The frequency of and potential change in medication intake, sleep, and diet during the pandemic was less clear, but there is virtually no evidence that the population as a whole improved their diabetes self-management during the COVID-19 pandemic.

Moving forward, researchers should aim to use standardized health behavior measures to allow for a comparison of findings across samples. In addition, where available, researchers should attempt to access pre-existing data, such as data located within patients’ medical records, to allow for more precision in their comparisons of health behavior prior to and during the pandemic. These data are especially important in identifying ways to support this population in returning to “normalcy” and re-establishing their diabetes self-management routines as the pandemic progresses and eventually ends. Even if the pandemic does eventually conclude, there may be other pandemics, natural disasters, climate disruptions, warfare, or civil unrest that have the potential to drastically impact the self-management of people with diabetes. In addition, researchers should prioritize conducting research with children and adolescents with T2DM. Moreover, future data should help identify those individuals with T2DM who demonstrated limited engagement in diabetes self-management behaviors pre-pandemic and continued to struggle, or deteriorate, throughout the pandemic. This subpopulation is important to study and may require more assistance in developing an individualized, specific diabetes self-management plan. Upon publication of further reports, a systematic review and meta-analysis should be conducted to further characterize the frequency of and changes in diabetes self-management health behaviors during the COVID-19 pandemic.

Clinically, providers are encouraged to closely monitor diabetes self-management in their patients, especially their older patients, as the pandemic continues to unfold globally with additional waves and new variants. Specifically, we recommend patient-provider problem-solving of specific steps to increase physical activity within the confines of COVID-19 guidelines and restrictions, such as scheduling daily 30-minute virtual physical activity exercises with friends or family. Patients should be encouraged to explore outdoor venues, such as parks, trails, and other open space, if available, in their neighborhood. Next, with the current increases in access to digital health technologies, healthcare providers should consider “prescribing” patients to download and complete digital health programs. This could include informal tracking of diet and physical activity with applications that allow users to share these data with friends and/or family, thereby providing external motivation and social support to improve the corresponding diabetes self-management behaviors. More structured digital health programs exist and should be considered. For example, see Fu et al. (70) for a review of existing diabetes/lifestyle applications. Last, and importantly, for those individuals with T2DM who demonstrate disruptions in diabetes self-management as a result of impaired psychological functioning in the context of the pandemic, providers should connect patients to psychological care where it is accessible. This might include “prescribing” patients mindfulness/meditation, relaxation, or other evidence-based digital mental health applications. In addition, provision of either in-person or telemedicine mental health services is recommended for patients experiencing significant mental health concerns and may elicit downstream improvements in diabetes self-management.

## Data Availability Statement

The data that support the findings of this study are available from the corresponding author, JW, upon reasonable request.

## Author Contributions

JW conceived of the study and wrote methods and results. KS screened articles. CC, JW, KS, and RT reviewed articles and populated tables. CC and JW wrote the manuscript. RT formatted tables. CC and KS designed figures. KS and RT edited the manuscript. All authors contributed to the article and approved the submitted version.

## Conflict of Interest

The authors declare that the research was conducted in the absence of any commercial or financial relationships that could be construed as a potential conflict of interest.

## Publisher’s Note

All claims expressed in this article are solely those of the authors and do not necessarily represent those of their affiliated organizations, or those of the publisher, the editors and the reviewers. Any product that may be evaluated in this article, or claim that may be made by its manufacturer, is not guaranteed or endorsed by the publisher.
